# Selected Proteins Involved in the Neuropathology of ASD as the Candidates for Fluid Biomarkers

**DOI:** 10.3390/molecules31111936

**Published:** 2026-06-03

**Authors:** Piotr Rutkowski, Adrianna Romanowicz, Jan Mroczko, Monika Gudowska-Sawczuk, Barbara Mroczko

**Affiliations:** 1 Mental Health Center for Children and Adolescents, University Children Clinical Hospital, 15-274 Bialystok, Poland; p.rutk@mp.pl; 2 Department of Neurodegeneration Diagnostics, Medical University of Bialystok, 15-269 Bialystok, Poland; adrianna.romanowicz@sd.umb.edu.pl (A.R.); mjanek2003@gmail.com (J.M.); 3 Department of Biochemical Diagnostics, Medical University of Bialystok, 15-269 Bialystok, Poland; monika.gudowska-sawczuk@umb.edu.pl

**Keywords:** autism, ASD, tau protein, NFL, BDNF, IGF-1

## Abstract

Autism spectrum disorder (ASD) is classified as a neurodevelopmental disorder with an increasingly high incidence rate. Increasing awareness, changing diagnostic criteria and social attitudes, as well as financial considerations, will affect the prevalence of diagnosis. The symptoms of ASD vary widely, making it difficult to detect. It represents a spectrum of alterations ranging from mild indications to severe impairments and diagnosis is based on a very rigorous behavioral assessment. Nevertheless, certain neuropathological changes are common, although the background of this disorder remains still unknown. Therefore, some research aimed at better understanding the pathology of the neurological alterations in ASD, as well as the possibilities for early diagnosis and treatment of this disorder, is urgently needed. This review summarizes the current evidence on some selected proteins such as tau protein, NFL, and BDNF as well as IGF-1 that appear to be the best protein candidates for better understanding the causes of autism, as well as for use as fluid biomarkers in the diagnosis and monitoring of ASD.

## 1. Introduction

Autism spectrum disorder (ASD) is a neurodevelopmental disorder that shapes the way people with autism perceive the world around them and interact with others [1]. In Europe, autism is diagnosed in approximately 1 in 100 children on average. Interestingly, the disorder occurs nearly three times more often in boys than in girls. People with autism develop differently from most, resulting in unique styles of communication and social interaction, and restricted, repetitive patterns of behavior, interests or activities. The symptoms of autism are extremely diverse and depend on each person’s individual characteristics, which is why this disorder is referred to as a spectrum. In some people, symptoms may be mild, while others may face serious difficulties in daily functioning. This situation requires an individualized approach in both diagnosis and therapy. Difficulties with processing sensory stimuli are another key aspect of autism. People with this disorder may be hypersensitive to sounds, light, or touch, which can lead to unpleasant or even painful sensations in everyday life.

The causes of autism are very complex and involve both genetic and environmental factors. Some investigations show that certain genetic abnormalities may increase the risk of developing this disorder. There are many gene mutations associated with brain development that may influence autism, but there is no single, specific gene responsible for its occurrence. Environmental factors also play a significant role, such as the parents’ age at conception, viral infections during pregnancy, and prematurity. Children born to older parents may be at greater risk of this disorder. We must not overlook the interactions between genes and the environment, which are also important in research on the causes of autism.

Currently, scientists are focusing on understanding how these various factors influence brain development and what mechanisms may lead to autistic symptoms. This complexity means that research is still ongoing, and experts are constantly searching for answers to better understand how different factors contribute to the onset of autism.

Early diagnosis of autism is extremely important because it allows for the implementation of effective support methods that can significantly improve the quality of life for people with this disorder. Many investigations on etiology, diagnosis, monitoring and treatment are based, in part, on biomarker studies. Nowadays, there are no specific biomarkers for autism diagnosis. However, the field of biomarker studies in ASD seems to be promising [2]. The ability to measure proteins as liquid biomarkers is particularly important when compared to neuroimaging studies. Blood and urine tests are less invasive and more accessible than other diagnostic methods. Furthermore, because these tests are performed on blood or urine, they might be used for screening, which is less invasive and less expensive compared to cerebrospinal fluid testing or neuroimaging methods.

Therefore, the aim of this paper is to compare the usefulness of selected candidates for biomarkers of this disease, such as tau protein, neurofilament light chain (NFL), and brain-derived neurotrophic factor (BDNF) as well as Insulin-Like Growth Factor 1 (IGF-1). Tau protein has been selected as the main factor in stabilizing axonal transport, while NFL has been chosen due to its specific presence in neurons as the major cytoskeletal protein. Moreover, BDNF is the main support of neuroplasticity, which is crucial for the processes of learning and remembering, while IGF-1 plays a dual role in neurodegenerative diseases—on the one hand, it has a neuroprotective effect, stimulating neuronal survival, neurogenesis and brain plasticity. On the other hand, disturbances in IGF-1 signaling accelerate the accumulation of toxic proteins and exacerbate inflammation. Therefore, the selection of these four proteins allows assessing the main areas related to ASD neuropathology.

This paper as a narrative review is based on the existing literature covering data on the possible role of the above-mentioned proteins in the pathophysiology of ASD as well as in the area of their usefulness as fluid biomarkers.

## 2. Tau Protein

The main function of tau protein is to maintain the proper structure and functioning of neurons. The most well-known role of the tau protein is to stabilize microtubules, ensuring the proper functioning of the neuronal cytoskeleton. In addition, tau protein participates in intracellular transport, enabling the movement of nutrients and organelles along axons. Under pathological conditions, it can undergo abnormal modifications, leading to its aggregation and nerve cell damage [3]. The most important post-translational modifications of tau proteins include their phosphorylation and dephosphorylation. As a result of the imbalance between phosphorylation and dephosphorylation processes, the number of highly phosphorylated isoforms of this protein (pTau) increases. An excessive phosphorylation causes the protein to lose its normal function, aggregate, and form toxic structures in neurons known as neurofibrillary tangles. This process leads to neuronal damage and cell death [4,5].

It is well known that tau proteins are strongly associated with neurodegenerative disorders. Diseases associated with Microtubule-Associated Protein Tau (MAPT) include primarily Alzheimer’s disease, but also frontotemporal dementia and corticobasal degeneration. Although tau protein is classically associated with neurodegenerative diseases, it also plays a role in brain development [6,7,8]. Therefore, its potential impact on neurodevelopmental disorders is increasingly being studied. Moreover, the link between tau protein and autism is investigated. Current research suggests that in some forms of autism spectrum disorders (ASDs), increased levels of tau protein may contribute to impaired neuronal function. However, it has been observed that the serum tau concentration in ASD children is significantly lower in comparison to healthy controls. Moreover, the levels of tau protein correlate with α-synuclein, a protein found abundantly in brain tissue and involved in the movement of synaptic vesicles and the release of neurotransmitters. The positive correlation may be due to the fact that both proteins influence the function and stability of neurons. Tau protein stabilizes microtubules, and α- synuclein regulates synapse function. Therefore, given that the neuron functions as an integrated whole, it appears that changes in the concentrations of one protein can directly affect the other [9]. Contradictory research results were obtained by H. Ayaydin et al., who reported higher serum tau levels in children with ASD than in a control group. It is difficult to clearly determine the reasons for these differences, but reduced tau levels in ASD may result from distinct mechanisms of regulation of neural development. These include altered expression or degradation of cytoskeletal proteins, reflecting the heterogeneity of neurobiological processes in autism. However, they also observed a significant correlation between tau protein and neuronal biomarkers, NSE and S100B, probably because all these three proteins are related to neuronal cell integrity and damage. Scientists also evaluated the diagnostic significance of tau protein in ASD. Based on the studies conducted, it seems that tau protein has potential diagnostic utility as a biomarker for autism spectrum disorder, but not as a specific marker. Its diagnostic performance is only moderate, with reported sensitivity (69.8%) and specificity (70.7%) values reflecting AUCs of 0.697, indicating limited discriminatory power. Therefore, its diagnostic value is limited and can only be considered as a complementary tool [10]. Interestingly, Öz et al. observed no significant differences in serum tau levels between the study group and the control group or healthy siblings. Furthermore, the tau levels did not significantly correlate with the severity of ASD symptoms. Given the inconsistent findings in the literature, it is difficult to definitively determine whether tau protein is a promising diagnostic biomarker for ASD [11].

As is well known, autism is classified as a neurodevelopmental disorder, and its first symptoms may appear as early as early childhood. People with autism spectrum disorder often struggle with building relationships, establishing contacts, and understanding social norms. Unfortunately, available treatments provide limited benefits. Consequently, new therapies are constantly being sought to improve patients’ quality of life. Tai et al. conducted studies in mice to determine whether genetically reducing tau protein prevents behavioral symptoms of autism. They found that partial or complete genetic removal of tau protein prevented the overactivation of the phosphatidylinositol 3-kinase (PI3K)/Akt (protein kinase B)/mTOR (mammalian target of rapamycin) signaling pathway. Tau protein interacts with phosphatase and tensin homolog deleted on chromosome 10 (PTEN) and weakens its inhibitory effect on the PI3K/Akt/mTOR pathway. Therefore, reducing tau levels “unblocks” PTEN, leading to the suppression of excessive activity in this pathway. Knowing that hyperactivation of this pathway increases synaptic activity and leads to an imbalance between excitation and inhibition in the brain, it can be concluded that reducing tau protein levels stabilizes neuronal activity. This reduces the excessive brain excitation often observed in ASD [12]. Mice with a mutation in the FMR1 gene, which encodes Fragile X Mental Retardation Protein (FMRP), crucial for normal neuronal development and function, were also studied. FMR1 and fragile X syndrome are strongly linked to autism spectrum disorders, as FMRP deficiency leads to impaired synaptic function and the development of neural networks. Tau expression was found to be increased in the cerebral cortex of FMR1 knockout mice. But reducing tau levels in FMR1 knockout mice led to significant behavioral improvements, reducing social deficits and repetitive behaviors, and partially normalizing other developmental abnormalities. Reducing tau also restored more normal activity of biological rhythms and partially corrected impaired Per1 gene expression, while reversing deficits in p38 MAPK signaling. This work shows that reducing tau protein levels in FMR1 mutant mice reduced autistic behaviors, suggesting that tau may be part of the mechanism of neuronal dysfunction in this model of autism spectrum disorder and indicating a potential avenue for research into new therapies [13]. Another experimental study examined whether mice with a deletion of B-integrin exhibited impaired excitability in the cerebral cortex. These disorders were found to occur, and the primary cause was excessive phosphorylation of tau protein. The abnormal regulation of this protein’s phosphorylation was dependent on the altered protein kinase A (PKA) pathway. The PKA pathway, like the above-mentioned pathways, is directly linked to tau protein, as PKA is also one of the kinases that can add phosphate groups to selected proteins. The altered tau phosphorylation status affected neuronal function, leading to abnormal activity and destabilization of neuronal networks in the cortex. In the B-integrin-deleted mouse model, PKA dysregulation occurs, leading to abnormal tau phosphorylation and impaired function of Ca^2+^-activated potassium channels (SK2) in neurons. As a result, neurons become excessively and unstably excitable, which may promote phenotypes resembling autism spectrum disorder [14].

Meanwhile, Dobrowolska et al. studied how tau protein changes in a model of autism induced by prenatal exposure to valproic acid (VPA). Observations revealed that “autistic” rats had abnormal levels of tau protein in the hippocampus and cerebral cortex. Furthermore, hyperactivity of the mechanistic target of rapamycin (mTOR) pathway and its associated kinases (CDK5, ERK1/2, and p70S6K) was demonstrated, leading to increased phosphorylation of tau protein in the hippocampus and cerebral cortex. This process results in tau dysfunction, neuronal alterations, and changes resembling autism spectrum disorder in the rat model. This was confirmed by immunohistochemical analysis, which revealed the presence of histopathological changes in neurons, including chromatolysis, in both studied brain regions in rats prenatally exposed to VPA [15].

A summary of tau protein findings is provided in Table 1.

In conclusion, tau protein plays a crucial role in maintaining the structure and function of neurons, stabilizing microtubules and participating in intracellular transport. Under pathological conditions, it undergoes abnormal modifications, particularly excessive phosphorylation, which leads to its aggregation, the formation of toxic structures, and nerve cell damage. It is strongly associated with neurodegenerative diseases such as Alzheimer’s disease, but its role in neurodevelopmental disorders, including ASD, is also increasingly being investigated. To date, research on the association of tau with ASD is equivocal—both elevated and decreased levels have been observed, and its diagnostic utility is limited. However, experimental studies in animal models suggest that reducing tau levels may improve neuronal function and reduce autism-like symptoms by affecting key signaling pathways in the brain. This indicates that tau protein may play a significant role in the mechanisms of neuronal disorders and represent a potential target for future therapies.

## 3. Neurofilament Light Chain (NFL)

Neurofilaments are neuron-specific structural proteins found in abundance in the axonal interior of healthy, myelinated neurons. They consist of subunits known as neurofilament light chain (NFL), medium chain (NFM), heavy chain (NFH), α-internexin and peripherin. Among the subunits, NFL is presently the most widely investigated as a biomarker in neurologic diseases. Under physiological conditions, a low amount of NFL is released during brain development, maturation and aging. It is observed in higher quantities in CSF and blood when axonal damage or neuronal degeneration occurs. It has been observed that it can be useful in several disorders such as neurodegenerative diseases, dementias, strokes or traumatic brain injuries [16,17].

The single-molecule array assay (SIMOA)-based detection of NFL is a very useful and sensitive method for diagnosing mild cognitive impairment (MCI) and Alzheimer’s disease (AD). Sahrai et al.’s [18] analysis showed—based on fifteen eligible studies including 3086 patients—that the NFL levels in plasma were significantly different between patients with MCI and AD. Elevated NFL levels have been associated with the presence of beta-amyloid plaques in pre-symptomatic individuals and with the level of tau protein in symptomatic patients [19]. Additionally, NFL levels in Parkinson’s diseases have been shown to correlate with disease severity and motor as well as cognitive decline [20]. NFL has been also widely studied as a marker of disease progression, treatment efficacy, and clinical outcomes for MS [21] and the concentrations of NFL are increased in early relapsing MS and have been shown to correlate with markers of disease severity. It is a biomarker of white matter axonal injury in patients with multiple sclerosis, revealing white matter changes in the course of follow-up studies [22]. What is worth emphasizing is white matter alterations have been indicated in ASD, and these abnormalities were correlated with changes in the functional connectivity [23]. Therefore, some studies have presented that NFL might be a potential biomarker connected with autism.

Öz et al. compared serum NfL (sNfL) levels in autism spectrum disorder (ASD) patients, their healthy siblings (HS), and healthy controls (HC). The aim of this study was to investigate the relationship of NFL levels with ASD severity [11] and the results of this paper indicated that sNfL levels in the ASD group were significantly higher than both of the control groups. Conclusions can be drawn based on these findings that NFL might be involved in the etiopathogenesis of ASD. However, the levels of neurofilaments have not been significantly associated with ASD symptom severity [11]. The results of the research of He et al. [24] were similar to those obtained by Öz et al. Two groups of scientists have shown that NfL concentrations were significantly higher in ASD than in the control group. However, the evidence provided in this study was not connected with the previous study in the category of the association between the severity of ASD and sNfL levels. In the paper of He et al., higher serum NfL levels were found to be correlated with both the elevated risk of ASD and the increase in the severity of ASD. The authors indicated that the Td size of the sample selection in these two papers (Öz and He), and the differences between the subjects who applied the scales and the clinical diagnosis of ASD, may explain the different correlations between ASD severity and NfL.

In a study by Simone et al. [25], sNfL and glial fibrillary acidic protein levels (GFAP) were investigated using SiMoA technology in a group of ASD patients and in a healthy control group (CTRS), age- and gender-matched. The comparison of serum Nfl and GFAP concentrations between ASD children and the control group revealed significantly higher levels in the ASD group. In this paper, it was also indicated that these biomarkers could be involved in both early diagnosis and etiological aspects in ASD. The authors concluded that the findings of their study might support the neuroinflammation and neurodegeneration hypothesis of ASD development and usefulness of these biomarkers in the early diagnosis of ASD as well as in the monitoring of the disease activity as well as in prognosis. Based on another study by Paketçi et al. [26], considering NFL and thrombospondin-1 levels, the concentration of these two proteins did not differ between study groups: ASD and typically developing children (TD). Stereotyped behavior and sensory sensitivity domain of the CARS scale (The Childhood Autism Rating Scale) were negatively correlated with serum TSP-1levels. Age was also positively correlated with NFL levels in the ASD groups but not in the TD group. This result was not connected with the previous study conducted by He and colleagues. The authors clarify that the mean age of the study of He was 5.1 years, which was lower than the mean age of the sample of Paketsi. It might partially explain the discrepancy between the results given and the significant correlation between age and serum NFL levels. Finally, the results of these investigations did not support the neurodegenerative processes in ASD, but for this, more studies with larger study groups are needed to indicate a link between NFL levels and the presence of ASD. A summary of NFL findings is provided in Table 2.

In conclusion, there are only a few studies on NFL usefulness in ASD for diagnosis, and no studies on follow-up and treatment connected with NFL concentrations. Therefore, additional studies, especially those accompanied by imaging analysis, are needed to fully understand the role of neurodegeneration and neuroaxonal damage in ASD based on sNfL.

## 4. Brain-Derived Neurotrophic Factor (BDNF)

Brain-derived neurotrophic factor (BDNF) belongs to the neurotrophin family, which also comprises nerve growth factor (NGF) and neurotrophins 3 and 4 (NT-3 and NT-4) [27]. It is widely expressed throughout the central nervous system (CNS) and functions as a key mediator of neuroplasticity. BDNF is critically involved in the regulation of neurogenesis and synaptogenesis, as well as in the survival, maintenance, and protection of diverse neuronal populations [28,29]. Moreover, it plays a central role in the induction of long-term potentiation (LTP), thereby contributing significantly to learning, memory formation, and higher cognitive processes [30,31,32]. BDNF is initially synthesized in the endoplasmic reticulum as a precursor protein (pre-pro-BDNF) and subsequently transported to the Golgi apparatus, where it is processed into pro-BDNF. Further proteolytic cleavage produces the mature isoform, known as m-BDNF [33]. A growing body of research has explored the link between BDNF and ASD in pediatric populations. The analysis conducted by Kasarpalkar et al. demonstrated that serum BDNF levels are distinctly correlated with the severity of clinical ASD symptoms. Markedly higher concentrations of this protein were observed in patients with a milder, atypical phenotype (*p* < 0.001), whereas no significant differences were found in the group with severe symptoms compared to the controls. An exception was noted in females with typical autism and Rett syndrome, who exhibited significantly lower concentrations of BDNF (*p* < 0.05). These findings allow for the interpretation of reduced BDNF serum levels as a deficit in neuroprotection, while high serum concentrations may represent a manifestation of compensatory mechanisms [34]. However, subsequent studies have yielded inconsistent results regarding the correlation between BDNF levels and symptom severity. Recent findings by Zhang et al. indicate that children aged 2–5 years with ASD show higher plasma BDNF concentrations and increased circulating oxidative stress compared to typically developing (TD) individuals. The elevated BDNF may reflect adaptive mechanisms related to atypical neurodevelopment, intensified synaptic pruning, or underlying inflammatory processes [35]. Contrary to the observations of milder phenotypes having higher levels, further research has demonstrated that BDNF concentrations are significantly elevated in the serum of children with autism (*p* < 0.001) and show a strong positive association with more severe symptoms as measured by the Childhood Autism Rating Scale (CARS) score. Interestingly, the introduction of omega-3 supplementation has been shown to markedly reduce these levels (*p* = 0.040), suggesting that omega-3 fatty acids may exert a protective effect against synaptic dysfunction in this population [36]. The complexity of BDNF’s role is further highlighted when considering older pediatric populations. A study involving children and adolescents aged 6–18 years found significantly lower BDNF serum levels in individuals with ASD compared to TD controls. This reduction was linked to heightened serum concentrations of the proinflammatory C-C motif chemokine ligand 5 (CCL5), indicating that chronic immune dysregulation can result in neuronal deficits. These decreased BDNF serum levels were directly associated with marked cognitive impairments and behavioral symptoms. In addition, executive function composite scores were negatively correlated with scores on the social interaction and communication subscales of the Autism Diagnostic Interview—Revised (ADI-R) [37]. 

Meng et al. reported elevated serum BDNF levels in a cohort of 82 children with ASD, demonstrating a significant negative correlation between BDNF concentrations and lower Intelligence Quotient (IQ) [38]. Similarly, Bryn et al. observed increased plasma BDNF levels in children with ASD compared to age- and sex-matched controls, with the highest levels noted in children with intellectual disability [39]. Moreover, in all studies reporting normal or reduced BDNF levels, individuals with ASD exhibited normal intellectual functioning or at least an IQ above 70 [40,41]. Peripheral BDNF is largely stored in platelets and released upon their degranulation, making platelet count a key factor influencing serum BDNF levels. In the study by Farmer et al., the initially higher BDNF concentrations observed in children with ASD were attenuated after adjusting for platelet count, indicating its role as an important confounding factor. This suggests that elevated BDNF serum levels in ASD may be partially attributable to increased platelet counts rather than solely reflecting neurobiological alterations. These findings highlight the importance of accounting for platelets in the analysis and interpretation of studies evaluating BDNF as a potential biomarker [42].

In conclusion, BDNF plays a central role in neuroplasticity, including neurogenesis, synaptogenesis, neuronal survival, and long-term potentiation. Increasing evidence suggests a link between BDNF and ASD; however, findings remain inconsistent. Some studies report elevated BDNF serum levels in children with ASD, associating them with symptom severity, oxidative stress, or compensatory neurodevelopmental mechanisms. Others indicate reduced serum levels, particularly in individuals with Rett syndrome and in older children, where the decline is associated with inflammatory processes, cognitive impairment, and behavioral symptoms. Additionally, both positive and negative correlations between BDNF and clinical features such as IQ or symptom severity have been observed. Importantly, as peripheral BDNF is largely stored in platelets, adjusting for platelet count leads to a reduction in measured BDNF levels and diminishes differences between ASD and controls, indicating its role as a key confounding factor. In addition, omega-3 supplementation has been demonstrated to lower abnormally elevated BDNF serum levels, which may help protect against synaptic dysfunction. These findings underscore the critical and multifaceted role of BDNF in the pathogenesis of ASD. A summary of the key findings regarding BDNF is presented in Table 3.

## 5. Insulin-like Growth Factor 1

Insulin-Like Growth Factor 1 (IGF-1) is a growth-promoting trophic factor that exerts its effects primarily through the IGF-1 receptor (IGF-1R), which is widely expressed across tissues, with particularly high levels in neuronal cells. Although most circulating IGF-1 is produced by the liver, it can cross into the brain, allowing peripheral IGF-1 to influence CNS levels [43,44]. IGF-1 expression in the brain is regulated mainly through two pathways. The first is autocrine/paracrine signaling, in which IGF-1 is locally produced by different brain cell types in a widespread manner across regions such as the cerebral cortex, hippocampus, cerebellum, and brainstem. The second is endocrine signaling, where IGF-1 from the circulation crosses the blood–brain and blood–cerebrospinal fluid (CSF) barriers to enter the brain and contribute to its local levels [45,46]. Emerging evidence suggests that IGF-1 plays a critical role in ASD pathogenesis.

Some studies have demonstrated that children with ASD may exhibit significantly elevated serum IGF-1 levels compared to (TD) peers [47,48]. Consistent with these findings, further research by Mashayekhi et al. has shown that both IGF-1 and IGF-2 concentrations are significantly higher in ASD patients than in controls. Notably, this serum IGF-1 increase appears to correlate with the progression of the disorder, with significantly higher concentrations observed across ASD stages I to III (representing mild to advanced clinical severity) [49]. In contrast, genetic analysis of the IGF-1 promoter polymorphism (rs12579108) revealed that individuals with AA genotype had reduced circulating serum IGF-1 levels and an increased susceptibility to ASD [50]. More recent findings indicate that children with ASD showed significantly lower serum IGF-1 levels compared to TD controls (*p* < 0.05). This reduction was especially evident in individuals with severe ASD, who also had significantly lower IGF-1 and Insulin-like Growth Factor-Binding Protein 3 (IGFBP-3) levels than those with mild-to-moderate symptoms (*p* < 0.001). A similar pattern was observed in the youngest subgroup (2–3 years), where concentrations of IGF-1 were significantly decreased in ASD compared to controls (*p* < 0.05). In addition, boys had significantly lower IGF-1 serum levels than girls in both the ASD and control groups (*p* < 0.05). Finally, IGF-1 and IGFBP-3 levels were negatively correlated with total CARS scores (r = −0.32 and r = −0.40, respectively; *p* < 0.001), indicating that lower serum levels were associated with greater symptom severity. To sum up, the reduction in the serum IGF-1 level in early childhood may be associated with the development of ASD [51]. A study by Yokoya et al. suggests that free IGF-1 in peripheral blood may be the primary source of urinary IGF-1 [52]. It means that urine levels could reflect the biologically active fraction of circulating serum IGF-1. In line with this, one study reported reduced urinary levels of IGF-1 and IGFBP-3 in children with ASD aged 2–5 years [53]. These findings are consistent with observations from other studies showing decreased IGF-1 in serum and CSF [51,54].

In addition, other studies have shown that various factors leading to maternal immune activation (MIA) are linked to an increased risk of ASD in offspring [55]. Children born to mothers experiencing MIA often display ASD-like behaviors, including repetitive actions and social impairments. In an MIA mouse model induced by prenatal infection, increased placental interleukin-6 (IL-6) expression was accompanied by reduced IGF-1 expression levels [56]. Notably, IGF-1 expression was restored in IL-6 knockout MIA mice, suggesting that upregulated IL-6 is responsible for suppressing IGF-1. Mechanistically, IL-6 has been shown to promote the interaction of suppressor of cytokine signaling 3 (SOCS3) with insulin receptor substrate-1 (IRS-1) or IGF-1R, thereby inhibiting IGF-1 signaling [57]. Consistently, increased expression of both IL-6 and SOCS3 has been observed in placentas from MIA models [56]. Overall, these findings suggest that reduced IGF-1 in MIA may result from IL-6-mediated inhibition via SOCS3.

In summary, it is undeniable that IGF-1 plays a key role in the pathogenesis of ASD, as demonstrated by the presented research findings. Moreover, the existing data demonstrate a high degree of heterogeneity. In early childhood, studies report both decreased and increased IGF-1 serum levels, suggesting variability that may depend on age, phenotype, or methodological differences. A similar inconsistency is observed in relation to symptom severity, where both elevated and reduced IGF-1 concentrations have been associated with different clinical stages and CARS scores. Genetic factors also appear to contribute, as specific polymorphisms (e.g., AA genotype of rs12579108) are linked to lower IGF-1 serum levels and increased ASD risk. In addition, prenatal MIA indicates that inflammatory pathways, particularly IL-6–SOCS3 signaling, may suppress IGF-1 expression during neurodevelopment. Table 4 provides a summary of findings on IGF-1 in ASD.

## 6. Summary

This review summarizes the most important information about selected proteins associated with ASD pathology, which appear to be promising candidates for early markers of developing neurological disorders leading to autism. These are the proteins associated with neuroinflammatory processes and neurodegenerative changes. Some of them, such as NFL and tau, are already used in the diagnosis and monitoring of certain neurological diseases as indicators of neuronal damage (NFL) or neurodegeneration (tau). Many studies point to their potential applications in ASD as well. A limitation of these studies is the difficulty in comparing groups across studies conducted at different centers (Table 5). Therefore, it is worth noting that certain factors influence test results, which may account for discrepancies in the findings. These include differences in patient age, disease severity, study methodology, as well as variations in the sensitivity and specificity of the methods used to measure proteins in biological fluids; for example, the assessment of NFL concentrations by ELISA method and using the SIMOA system is not always comparable. An additional cause of inconsistent test results may be changes in brain activity over the course of a day. Tau levels naturally fluctuate according to the circadian rhythm. During wakefulness, active neurons release certain amounts of tau protein. If the body does not enter a state of sleep, this protein is not cleared efficiently and begins to accumulate. Therefore, the levels of tau proteins depend on the quality and length of sleep. It is not always taken into account in the assessment of the study group. Currently, there is no single protein-specific factor for ASD diagnosis. However, GI microbial metabolites in the urine are, in fact, effective biomarkers for ASD [58,59]. On the other hand, a thorough understanding of the causes of the pathology leading to ASD is crucial for implementing early interventions to prevent the development of this increasingly common disorder. Therefore, further studies on larger groups are needed to enable the specific proteins to be used in clinical practice.

## Figures and Tables

**Table 1:** Summary of research on tau protein in ASD.

Study Type	Tau Protein Concentration	Main Conclusion	References
Clinical study (ASD vs. control)	↑	Inconsistent results, possible heterogeneity of ASD groups	[10]
	↓		[9]
	No significant change		[11]
Correlations	α-synuclein	Tau protein is associated with other proteins	[9]
	NSE, S100B		[10]
Animal models	Altered phosphorylation usually ↑	Tau protein is involved in neuronal dysfunction in ASD animal models, with alterations in its expression and phosphorylation driven by dysregulated signaling pathways (e.g., PI3K/Akt/mTOR and PKA)	[12,13,14]
Diagnostic significance		Tau protein has limited diagnostic value (non-specific marker)	[10]

**Table 2:** Studies of NFL measurements in ASD.

Study Groups	NFL Concentration	Main Conclusion	References
ASD children and healthy control group (CTRS)	↑ (elevated)	The comparison of NFL serum levels between ASD children and the control group showed a mean value of these two markers significantly higher in the ASD group	[25]
Autism spectrum disorder (ASD) patients, their healthy siblings (HS), and healthy controls (HC)	↑ (elevated)	sNfL levels in the ASD group were significantly higher than both of the control groups	[11]
Individuals with autism and typically developing (TD) children	↑ (elevated)	Increases in serum NfL levels were found to be correlated with both the higher risk of ASD and the increase in the severity of ASD in this study	[24]
ASD and typically developing children (TD)	No significant change	The concentration of NFL did not differ between study groups: ASD and typically developing children	[26]

**Table 3:** Summary of findings on BDNF in ASD.

Study Type	BDNF Concentration	Main Conclusions	References
Early Childhood Studies (ASD vs. TD)	↑	In children aged 2–5 years, serum concentration of BDNF is typically higher than in typically developing (TD) peers. This is linked to atypical neurodevelopment, oxidative stress, and intense synaptic pruning.	[35,42]
Adolescent (ASD vs. TD)	↓	In older groups (6–18 years), BDNF serum levels are often lower than in TD controls, reflecting chronic immune dysregulation (high CCL5 concentrations) and exhausted neuroprotection.	[37]
Cognitive Correlation (IQ)	↓ in lower IQ	Strong negative correlation: the most significant elevations of serum are found in children with intellectual disability. Patients with IQ > 70 often show normal or reduced serum levels.	[38,39]
Symptom Severity (CARS)	↓ or ↑	Findings are inconsistent: some data indicate a positive correlation between high BDNF serum levels and severe symptoms (CARS), whereas others suggest that elevated serum levels of BDNF in milder phenotypes act as a compensatory mechanism that fails in more severe cases.	[34,36]
Biological Factors (Platelets)	↑	Elevated serum BDNF levels may be partially explained by higher platelet counts in ASD children. Platelets are the primary peripheral storage site for BDNF.	[42]
Gender & Specific Syndromes	↓	Significantly lower concentrations are observed in females with typical autism and individuals with Rett syndrome.	[34]
Therapeutic Impact (Omega-3)	↓ (Normalization)	Omega-3 supplementation has been shown to reduce pathologically high BDNF levels, potentially protecting against synaptic dysfunction.	[36]

**Table 4:** Summary of findings on IGF-1 in ASD.

Study Type	IGF-1 Concentration/Expression	Main Conclusions	References
Early Childhood Studies (ASD vs. TD)	↑ or ↓ (Divergent)	Many studies show decreased serum and urinary levels in children aged 2–5. Conversely, these results contrast with reports of elevated concentrations of IGF-1, highlighting the high heterogeneity.	[47,51,53]
Symptom Severity (Stages I–III/CARS)	↑ or ↓ (Divergent)	Higher IGF-1 serum levels have been noted in advanced clinical stages, whereas other studies found that lower concentrations were associated with greater symptom severity on the CARS scale.	[49,51]
Genetic Factors (Polymorphism)	↓ in AA genotype	Individuals with the AA genotype (rs12579108) exhibit significantly reduced serum IGF-1 levels and an increased susceptibility to ASD compared to CC/CA genotypes.	[50]
Prenatal Factors (MIA Model)	↓ Reduced expression	MIA leads to upregulated IL-6, which triggers SOCS3 to inhibit IGF-1 signaling, resulting in reduced IGF-1 expression during fetal development.	[56,57]

**Table 5:** Comparative overview of selected ASD biomarker candidates.

Protein	Sample Type Used in ASD Studies	Age Group Relevance	Key Confounding & Influencing Factors	Diagnostic Accuracy
Tau protein	Primarily serum in clinical studies, brain tissue in animal models [9,10]	No explicit pediatric age cohorts specified in the text.	Circadian rhythm: Natural daily fluctuations in protein levels. Sleep ecology: Insufficient clearance and protein accumulation due to poor quality and length of sleep.	Diagnostic sensitivity (69.8%) and specificity (70.7%) values reflecting AUCs of 0.697 (serum) [10].
NFL	Plasma, serum [11,18]	Physiological release depends on brain development, maturation, and aging. Positive correlation between age and NFL levels in older pediatric samples [26].	Physiological maturation and age.	Not available.
BDNF	Plasma, serum [35,37]	Early childhood (2–5 years): Elevated plasma concentrations of BDNF [35]. Older pediatric populations (6–18 years): Significantly decreased serum levels of BDNF [37].	Platelet count: Major confounding factor diminishing group differences after statistical adjustment [42]. Omega-3 supplementation: Marked reduction in pathologically elevated levels [36]. Chronic immune dysregulation: Significant co-occurrence of pathologically high serum levels of proinflammatory chemokine CCL5 with decreased BDNF serum levels in older children [37].	Not available.
IGF-1	Serum, urine [51,53]	Youngest subgroup: Significantly decreased serum of IGF-1 concentrations [51]. Early childhood: Reduced urinary levels of IGF-1 [53].	Gender: Significantly lower serum levels in boys than in girls [51]. Genetics: Reduced serum levels in individuals with the rs12579108 AA genotype [50].	Not available.

## Data Availability

No new data were created or analyzed in this study.
